# Human Proximal Tubule Epithelial Cells (HK-2) as a Sensitive In Vitro System for Ochratoxin A Induced Oxidative Stress

**DOI:** 10.3390/toxins13110787

**Published:** 2021-11-06

**Authors:** Enrique García-Pérez, Dojin Ryu, Hwa-Young Kim, Hae Dun Kim, Hyun Jung Lee

**Affiliations:** 1 School of Food Science, Washington State University, P.O. Box 646376, Pullman, Washington, DC 99164-6376, USA; enrique.gape@gmail.com; 2 Department of Animal, Veterinary, and Food Sciences, University of Idaho, 875 Perimeter Drive MS 2330, Moscow, ID 83844-2330, USA; dryu@uidaho.edu (D.R.); haedunk@uidaho.edu (H.D.K.); 3 Department of Biochemistry and Molecular Biology, Yeungnam University College of Medicine, Daegu 42415, Korea; hykim@ynu.ac.kr

**Keywords:** ochratoxin A (OTA), oxidative stress, renal carcinogen, kidney cell lines, LLC-PK1, HK-2

## Abstract

Ochratoxin A (OTA) is a mycotoxin that is potentially carcinogenic to humans. Although its mechanism remains unclear, oxidative stress has been recognized as a plausible cause for the potent renal carcinogenicity observed in experimental animals. The effect of OTA on oxidative stress parameters in two cell lines of LLC-PK1 and HK-2 derived from the kidneys of pig and human, respectively, were investigated and compared. We found that the cytotoxicity of OTA on LLC-PK1 and HK-2 cells was dose- and time-dependent in both cell lines. Furthermore, increased intracellular reactive oxygen species (ROS) induced by OTA in both cell lines were observed in a time-dependent manner. Glutathione (GSH) was depleted by OTA at >48 h in HK-2 but not in LLC-PK1 cells. While the mRNA levels of glucose-6-phosphate dehydrogenase (G6PD) and glutathione peroxidase 1 (GPX1) in LLC-PK1 were down-regulated by 0.67- and 0.66-fold, respectively, those of catalase (CAT), glutathione reductase (GSR), and superoxide dismutase 1 (SOD) in HK-2 were up-regulated by 2.20-, 2.24-, and 2.75-fold, respectively, after 72 h exposure to OTA. Based on these results, we conclude that HK-2 cells are more sensitive to OTA-mediated toxicity than LLC-PK1, and OTA can cause a significant oxidative stress in HK-2 as indicated by changes in the parameter evaluated.

## 1. Introduction

Ochratoxin A (OTA) is a potent mycotoxin that is a possible human carcinogen classified in Group 2B by the International Agency for Research on Cancer (IARC) [1]. Due to the different growth requirements of the fungal species that produce OTA in the genera of *Aspergillus* and *Penicillium*, OTA can be found in a wide variety of agricultural commodities and their processed products, including cereal grains, coffee, nuts, and wine [2,3,4]. While epidemiological data are lacking, the array of toxicities in animals associated with dietary exposure to OTA suggests OTA as a public health concern. OTA is well known for its kidney toxicity in different animal species, and it causes kidney tumors in rodents [5,6]. OTA is also known to be hepatotoxic, teratogenic, mutagenic, and immunosuppressive [7,8,9].

The exact mechanism of OTA toxicity or chemical carcinogenesis has not been elucidated yet. According to the proposed mechanisms related to OTA toxicity, acute and chronic toxicity of OTA are related directly or indirectly to (a) inhibition of mitochondrial respiration and ATP production [10,11]; (b) inhibition of protein synthesis [12,13]; (c) OTA-induced DNA damage [14,15]; (d) lipid peroxidation [16,17,18]; and (e) the production of reactive oxygen species (ROS) and resulting oxidative stress [19,20,21]. Based on the toxicological data available to date, oxidative stress appears to be the most plausible underlying mechanism of toxicity of OTA [22,23]. However, the involvement of oxidative stress in OTA-mediated toxicity remains debatable, and several reports concluded that oxidative stress does not play a pivotal part in OTA-mediated toxicity [24,25,26]. The occurrence of oxidative stress/damage is worth confirming, as several studies reported varying oxidative (DNA) damage in the kidney in vitro and in vivo [14,20,27,28,29].

OTA’s half-life in animals and human is 77 h in young calves [30], 88.8 h in pigs, 8.2 h in rabbits, 4.1 h in chickens [31], and 35.6 days in humans [32]. More importantly, differences in the OTA serum/plasma half-life account for most of the high variability in OTA toxicity observed among different animal species. While pig is generally considered as being the most sensitive to OTA nephrotoxicity among animal species [33], it is unclear whether other species may be more sensitive, particularly in responding to OTA-induced oxidative stress. Thus, this study aimed to examine and compare OTA-mediated oxidative stress in two immortalized cell lines, i.e., the kidney proximal tubule epithelial cell lines derived from pig and human kidneys, LLC-PK1 and HK-2, respectively.

## 2. Results

### 2.1. Effect of OTA on Cell Viability

**Figure 1:**
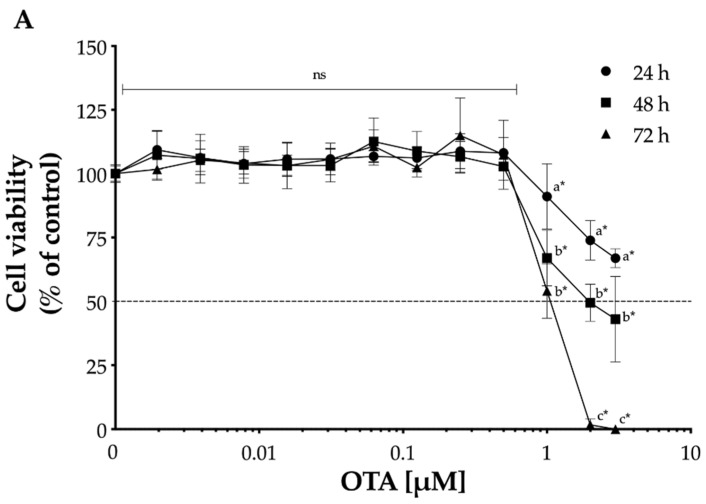

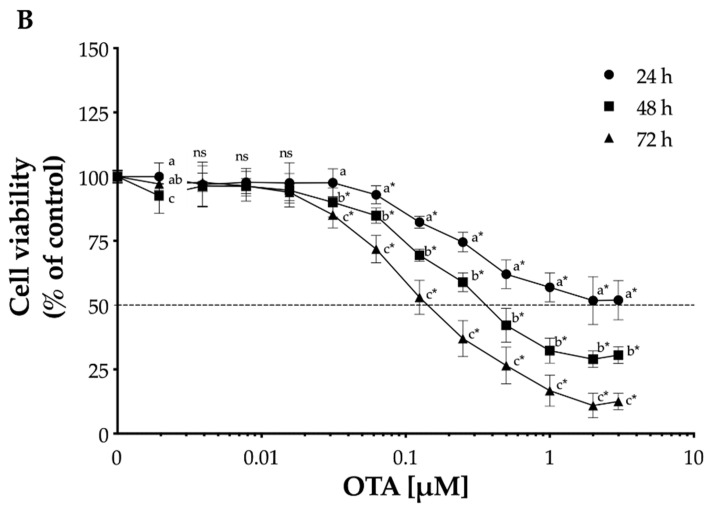
Time- and concentration-dependent cytotoxicity of ochratoxin A (OTA) in proximal tubule epithelial cells. Cell viability was determined by the reduction in MTT after incubation with OTA for 24 h (•), 48 h (■), and 72 h (▲) in LLC-PK1 (**A**) and HK-2 (**B**) cells. Values are mean (*n* = 3) ± standard deviation, and they are expressed as percentage of control. The dotted line (···) represents the concentration of OTA that reduced the cell viability by 50%. Different letters indicate a statistically significant difference (*p* < 0.05) between values within the same OTA concentration by Tukey’s multiple comparison test, while ns = non-significant. Asterisks (*) indicate a statistically significant difference (*p* < 0.05) between values against each group control by Dunnett’s test.

To determine the effect of OTA on cell viability, LLC-PK1 and HK-2 cells were treated with various concentrations of OTA, ranging from 1.95 nM to 3 μM, for different time periods (24 h, 48 h, and 72 h). In both cell lines, OTA led to a dose- and time-dependent decrease in cell viability (Figure 1). After 24 h OTA exposure, statistically significant (*p* < 0.05) effects were noted at 1 μM and 31.25 nM for LLC-PK1 and HK-2, respectively. Based on the dose- and time-dependent cell viability curves, the IC_50_ values for each OTA exposure time on LLC-PK1 and HK-2 cells (Table 1) were calculated. The effect of OTA on cell viability under serum-free conditions was evaluated in this study, as OTA binds to albumin [34], and thus, the presence of serum in the culture medium has been shown to decrease OTA cytotoxicity in both LLC-PK1 and HK-2 cells [21,35,36,37,38,39,40,41].

### 2.2. Effect of OTA on Cellular Redox State

**Figure 2:**
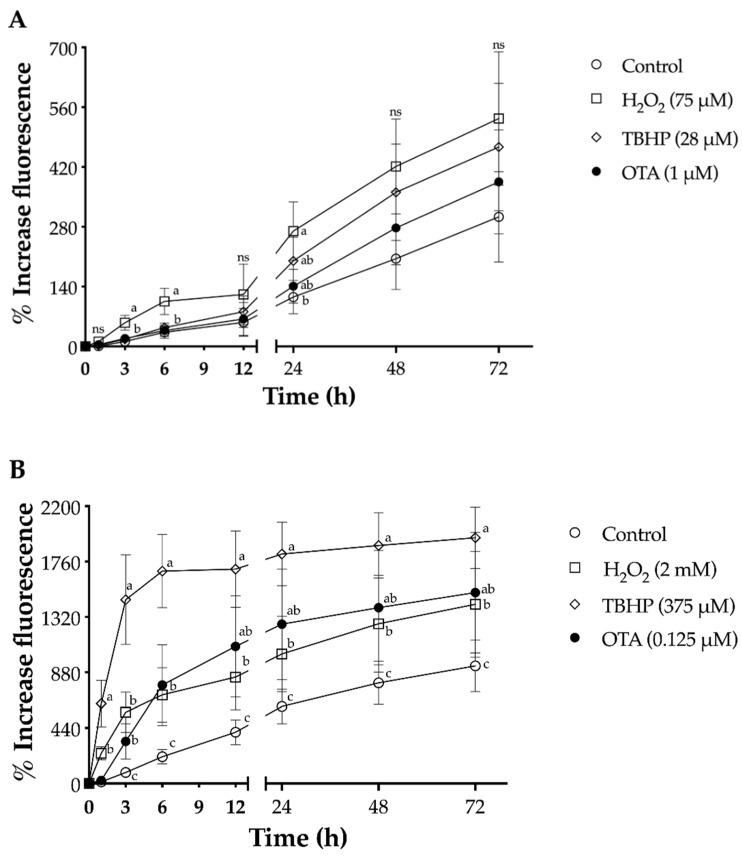
Production of reactive oxygen species by H_2_O_2_, TBHP, and ochratoxin A (OTA) in proximal tubule epithelial cells at various exposure times up to 72 h. Increase in DCF fluorescence, upon oxidation of carboxy-H_2_DCF, was monitored after exposure to the test compound. (**A**) LLC-PK1 cells H_2_O_2_ (☐ ,75 μM), TBHP (△, 28 μM), and OTA (•, 1 μM); (**B**) HK-2 H_2_O_2_ (☐, 2 mM), TBHP (△, 375 μM), and OTA (•, 0.125 μM). In both cell lines, non-treated controls are denoted by open circles (◯). Values are mean (*n* = 3) ± standard deviation, and they are expressed as percentage increase in fluorescence from time zero. Different letters indicate a significant difference (*p* < 0.05) between treatments at each time by Tukey’s multiple comparison test, while ns = non-significant difference.

A plausible mechanism by which OTA exert its toxicity is through the development of oxidative stress. Thus, OTA-mediated ROS formation was investigated through the oxidation of carboxy-H_2_DCF (dichlorodihydrofluorescein) to DCF (dichlorofluorescein). Figure 2 shows that OTA induced ROS in a time-dependent manner and that the concentration of ROS formed was higher in HK-2 compared with that in LLC-PK1 cells. After OTA exposure in both cell lines, the first significant (*p* < 0.05) effect was noted at 48 h and 3 h for LLC-PK1 and HK-2, respectively. In LLC-PK1 cells, formation of ROS at an elevated level was observed after 24 h exposure, but the OTA concentrations evaluated were higher compared with the concentration used in our study [21,42,43]. On the other hand, higher concentration of OTA (up to 800 μM) in HK-2 cells has been shown to increase ROS by 20% above control, whereas 50 μM OTA only increased ROS by 10% after 24 h exposure [39,44]. In contrast, these results indicate that 0.125 μM OTA was able to increase ROS levels significantly (*p* < 0.05) by 3.8-fold compared with control after 3 h OTA exposure.

### 2.3. Effect of OTA on Intracellular GSH Levels

**Figure 3:**
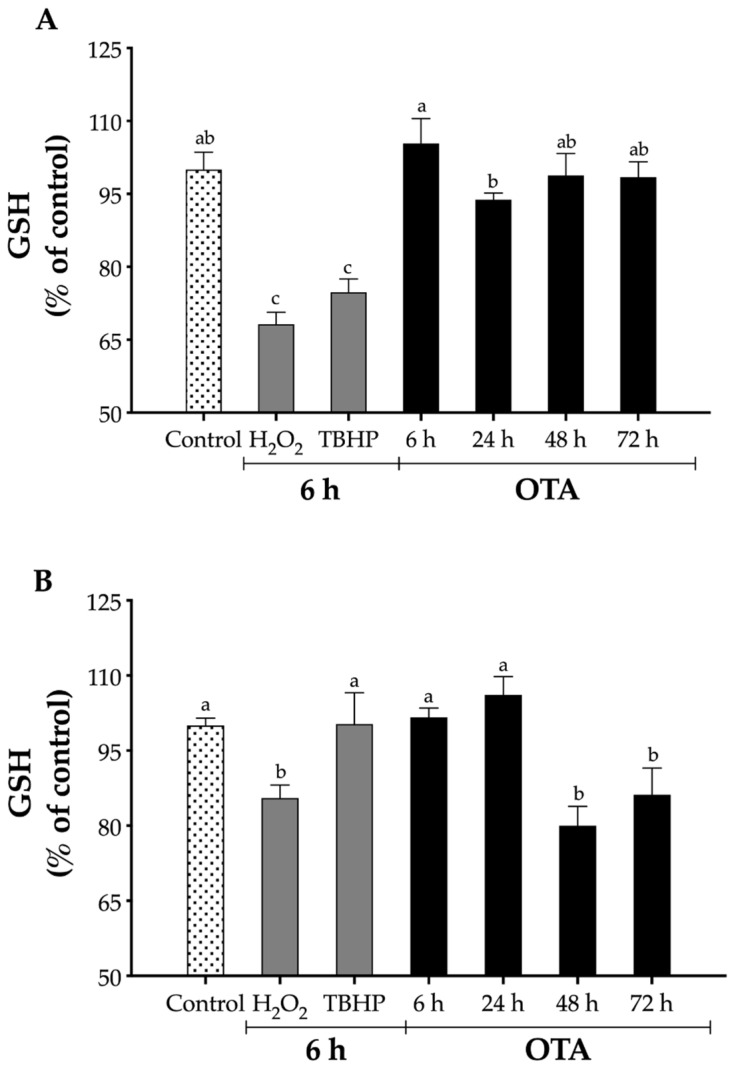
Relative levels of GSH in proximal tubule epithelial cells. GSH levels were determined by the reaction with DTBN to form 5′-thio-2-nitrobenzoic acid in cells homogenates. (**A**) LLC-PK1 cells H_2_O_2_ (75 μM), TBHP (28 μM), and OTA (1 μM); (**B**) HK-2 H_2_O_2_ (2000 μM), TBHP (375 μM), and OTA (0.125 μM). Values are mean (*n* = 3) ± standard deviation, and they are expressed as percentage of control. Different letters indicate a statistically significant difference (*p* < 0.05) between treatments at each time by Tukey’s multiple comparison test.

Varying responses of the two cell lines to OTA were investigated by measuring intracellular GSH (reduced form) levels (Figure 3). In LLC-PK1 cells, a statistically significant (*p* < 0.05) reduction in GSH levels was observed only when the cells were exposed to either H_2_O_2_ and TBHP by 32% and 25%, respectively, although OTA did not affect the levels of GSH at any time (Figure 3A). In contrast, HK-2 cells were affected only by H_2_O_2_ and OTA at 48 h and 72 h, at which time GSH levels were reduced by 15%, 20%, and 15%, respectively (Figure 3B).

### 2.4. Effect of OTA on Antioxidant Enzymes Gene Expression

**Figure 4:**
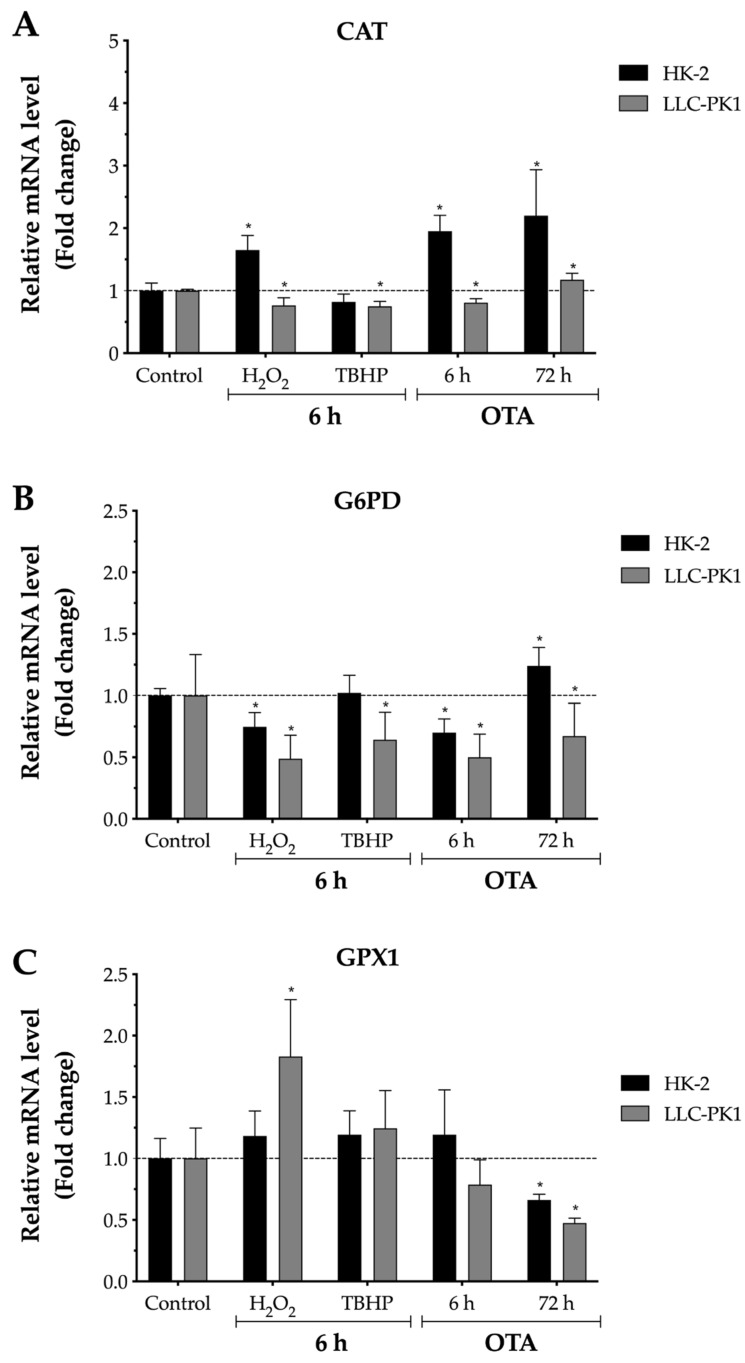

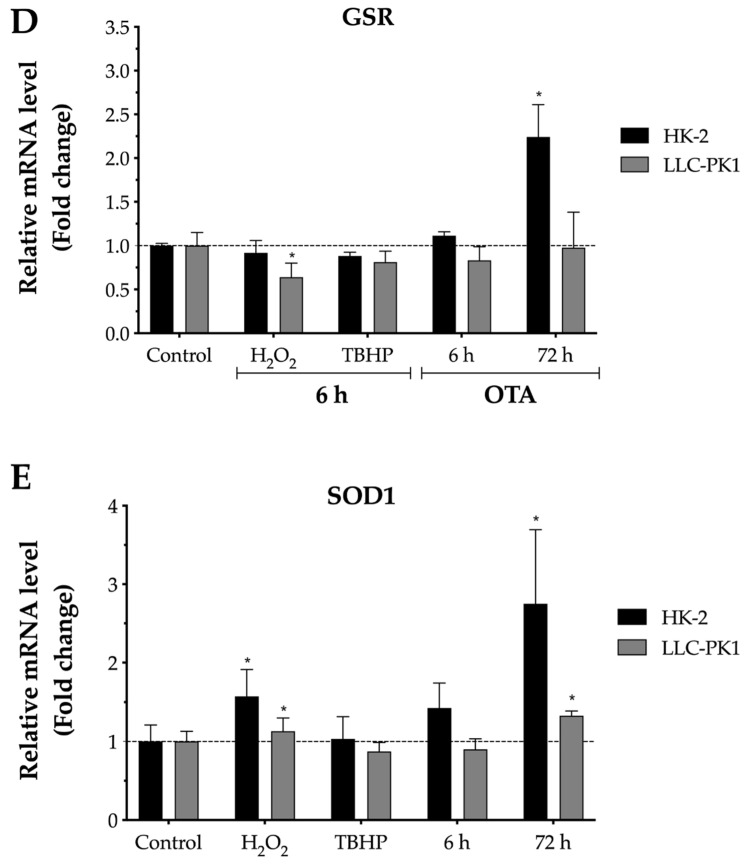
Relative mRNA levels of CAT, G6PD, GSR, GPX, and SOD in proximal tubule epithelial cells after exposure to H_2_O_2_, TBHP, and ochratoxin A (OTA). The mRNA expression of each gene was normalized using GADPH mRNA expression as housekeeping gene. LLC-PK1 cells H_2_O_2_ (75 μM), TBHP (28 μM), and OTA (1 μM). HK-2 H_2_O_2_ (2 mM), TBHP (375 μM), and OTA (0.125 μM). (**A**) Catalase, CAT. (**B**) Glucose-6-phosphate dehydrogenase, G6PD. (**C**) Glutathione peroxidase 1, GPX1. (**D**) Glutathione reductase, GSR. (**E**) Superoxide dismutase 1, SOD1. Values are mean (*n* = 3) ± standard error of the mean, and they are expressed as fold change by the 2^−ΔΔCt^ method. The dotted line (···) represents the level of mRNA for control cells. Asterisks (*) indicate a statistically significant difference (*p* < 0.05) between values against each cell line control by Student’s t-test. The fold change in OTA 72 h group was normalized with an independent control at 72 h.

To further understand the differential involvement of OTA on oxidative stress in the two cell lines, responses to OTA were measured with the mRNA expression levels of CAT, G6PD, GPX1, GSR, and SOD1, which are key enzymes in the detoxification of ROS (Figure 4). The result was considered biologically meaningful when the mRNA levels changed ≥1.5-fold that of control, either down- or up-regulated [45]. In LLC-PK1 cells, G6PD was down-regulated at 6 h and 72 h after OTA exposure (0.5- and 0.67-fold, respectively), while GPX was affected by OTA only after 72 h (0.66-fold) compared with control (Figure 4B–C). On the other hand, in HK-2 cells, only GPX was down-regulated at 72 h after OTA treatment (0.66-fold), whereas CAT, GSR, and SOD mRNA levels were up-regulated after 72 h incubation with OTA (2.20-, 2.24-, and 2.75-fold, respectively) compared with control (Figure 4A, 4D, 4E). The up-regulation of these genes is in line with our results showing an increase in ROS levels (Figure 2B) and a decrease in GSH levels (Figure 3B), as CAT and SOD neutralize H_2_O_2_ and O_2_•–, respectively, while GSR reduced GSSG to maintain GSH. These antioxidant enzymes are known to be up-regulated during oxidative stress to counteract its toxic effects [46].

## 3. Discussion

The cytotoxicity of OTA on LLC-PK1 and HK-2 cells observed after 24 h exposure agreed with previous studies [35,39]. It is noteworthy to mention that the OTA concentrations did not exceed 3 μM, as OTA solubility in water at 25 °C has been reported to be between 1.05 μM and 3.25 μM [47,48]. Under the conditions evaluated in this study, HK-2 cells were more sensitive to OTA-mediated cytotoxicity, as determined by the IC_50_ value (Table 1), which is consistent with other reports indicating differences in OTA-sensitivity among several species [38,49]. Jennings et al. [50] investigated the effect of OTA on three human renal proximal tubular models (HK-2, RPTEC/TERT1, and human primary cells), two rat renal proximal tubular models (NRK-52E and rat primary cells), and Wistar rat *in* vivo model. The study determined the largest differences between the human and rat cell culture models, while the results from human cell lines showed more similarity to the in vivo rat model because it is more likely that these differences are model-specific rather than species-specific per se [50]. The reason that human kidney cells are more sensitive to OTA toxicity may be explained by the differences in OTA uptake, differential OTA binding to intracellular proteins, and OTA elimination half-life. The H^+^-dipeptide cotransport is involved in the uptake of OTA in Madin-Darby canine kidney cells (MDCK), but it is not expressed in LLC-PK1 [51,52,53]; however, this transporter has not been studied in HK-2 cells. While LLC-PK1 is a spontaneously immortalized cell line, HK-2 is immortalized by transduction with human papilloma virus 16 (HPV-16) E6/E7 genes. The E6/E7 genes of HPV-16 have been reported to immortalize epithelial cells of diverse origin without significantly changing its phenotype or function [54,55,56,57,58,59]; however, most of immortalized cell lines have potentially limited their utility with derangements in cell function and structural integrity as a result of altered gene expression [60,61,62,63]. In addition, differences in OTA binding to intracellular proteins have been observed among several species, where human kidney homogenates showed a higher OTA protein binding than pig kidney homogenates [64,65,66]. This observation may also explain the differences in OTA-mediated cytotoxicity in this study, but these proteins have not been identified yet. Moreover, differences in elimination half-life of OTA varies greatly among species, e.g., 840 h in humans, 72–120 h in pigs, and 55–120 h in rodents, which may also influence the OTA-mediated toxicity in vitro [31,32,67,68].

ROS, such as superoxide anion (O2•−), hydroxyl radical (OH•), and hydrogen peroxide (H_2_O_2_) are produced during aerobic cellular metabolic processes. Although ROS play an important role in cell homeostasis, they can also promote the development of oxidative stress [69]. On one hand, ROS are required for the regulation of several physiological mechanisms, such as cell differentiation, cell proliferation, apoptosis, and regulation of redox sensitive signal transduction pathways [69]. At the same time, increased levels of ROS in the cell can also result in damage including cell death, mutations, chromosomal aberrations, and carcinogenesis [69]. Therefore, intracellular concentration of ROS is controlled by its production and/or removal by antioxidant systems. When the detoxifying mechanism fails, either by its inhibition or because it is not enough to counteract ROS levels, the cells undergo oxidative stress. It is important to mention that the oxidation of H_2_DCF to DCF is not specific for any particular ROS, and it also indicates the impact of OH• formed during Fenton-type reactions between H_2_O_2_ and Fe^2+^ [70]. In addition, it has been observed that cytochrome *c* is able to oxidize H_2_DCF [70]. In line with this, OTA has been shown to induce apoptosis through caspase-3, in both human and pig renal cells, which is activated by cytochrome *c* once released from the mitochondria upon mitochondrial membrane permeabilization [36,71,72,73,74,75]. Thus, OTA may be contributing to the increase in ROS formation via indirect mechanisms.

Oxidative stress produced by H_2_O_2_ and TBHP is well documented. The degree of ROS production by H_2_O_2_ and TBHP in LLC-PK1 and HK-2 by time was shown to be different in this study (Figure 2), due to their different mechanism in the body system. TBHP, which is a toxic compound, can cause extreme discomfort to body systems, including various organs, when it is exposed to cellular components by increasing membrane permeability along with hyperpolarization [76,77,78]. *t*-Butoxyl radicals that initiate lipid peroxidation are formed by reaction between TBHP and hemoglobin, and then extensive lipid peroxidative leads to membrane disturbance [79,80]. Therefore, cellular antioxidant systems such as GSH inhibit membrane disruption by scavenging the *t*-butoxyl radicals. H_2_O_2_, which is one of the major ROS, is known as an inducer necrosis-dependent on poly (ADP-ribose) polymerase-1 (PAPR-1), and a large amount of exogenous H_2_O_2_ causes oncotic death in cultured endothelial cells [81].

The active antioxidant system against ROS includes low molecular and high molecular mass antioxidants [46]. The most important endogenous antioxidant is GSH, as it is used to control ROS levels either directly (e.g., reaction with O_2_•−), or serving as a cofactor for ROS-detoxifying enzymes (e.g., glutathione peroxidase). High molecular mass antioxidants include antioxidant enzymes, such as superoxide dismutase (SOD), catalase (CAT), and glutathione peroxidase (GPX). SOD converts O_2_•− to molecular oxygen (O_2_) and H_2_O_2_, CAT converts H_2_O_2_ into oxygen and water, and GPX converts H_2_O_2_ into water [12]. In addition, GPX requires GSR and G6PD for its activity, which regenerates GSH and reduces nicotinamide adenine dinucleotide phosphate (NADPH) from its oxidized counterparts—glutathione disulfide (GSSG) and NADP^+^, respectively [46]. Endogenous antioxidants play an important role in neutralizing ROS, and among them, GSH is considered the most abundant molecule (1–10 mM) [82]. GSH can scavenge ROS either directly (e.g., reaction with O_2_•–) or serving as a cofactor for ROS-detoxifying enzymes (e.g., glutathione peroxidase). While a significant (*p* < 0.05) reduction in GSH levels in LLC-PK1 cells was observed when cells were exposed to either H_2_O_2_ or TBHP, OTA did not affect the levels of GSH at any time (Figure 3A). This agrees with other studies in which OTA did not decrease the levels of GSH when up to 10 μM OTA were tested in pig renal cells (LLC-PK1 and PK15 cells) after 24 h incubation [21,83]. Nonetheless, GSH depletion was observed when LLC-PK1 cells were exposed to 100 μM OTA for 24 h, which was concomitant with the high ROS level detected [21]. In contrast, HK-2 cells were affected only by H_2_O_2_ and OTA at 48 h and 72 h (Figure 3B). It has been reported that OTA does not decrease GSH levels in human embryonic kidney cells (HEK293) after 24 h and 48 h incubation with OTA [84]. Thus, based on our results in HK-2 cells, the depletion of GSH is concomitant with the high levels of ROS observed (Figure 2B). To our knowledge, this is the first study conducted to evaluate the effect of OTA on GSH levels in HK-2 cells.

G6PD is an enzyme that maintains the levels of NADPH, which in turn promotes GSH regeneration; thereby, cells are protected against oxidative damage and injury [85]. Some G6PD transcription suppressors include cyclic adenosine monophosphate (cAMP) response element modulator (CREM), tumor necrosis factor alpha, and tumor suppressor p53 [86]. OTA has been shown to activate p53 in both monkey and human kidney cells, Vero and HEK293, respectively, and thus, the down-regulation of G6PD after OTA treatment in LLC-PK1 cells may be explained by the activation of p53 [87]. GPX1 is a selenium-dependent enzyme that utilizes GSH as a co-factor to detoxify H_2_O_2_ and other organic peroxides [46]. It is known that GPX1 activity and protein and mRNA levels decrease exponentially in progressive selenium deficiency. Recently, it was reported that the supplementation of selenomethionine alleviates OTA-induced toxicity in porcine renal (PK15) cells by enhancing GPX1 expression [88,89].

## 4. Conclusions

The most important finding of this study was that OTA induces different response in both porcine and human proximal tubule cells (LLC-PK1 and HK-2 cells, respectively). HK-2 cells at nanomolar OTA concentrations were more susceptible to OTA-mediated cytotoxicity than LLC-PK1 cells. Although some hypotheses have been proposed to explain the differences in cytotoxicity among several species, the exact mechanism is not well understood. In line with the decrease in cell viability, increased ROS levels as well as depletion of GSH in HK-2 cells were observed, while these effects were not observed in LLC-PK1 cells. Therefore, future work may attempt to understand whether OTA increases ROS levels by direct redox-cycling of OTA or by indirectly relocating lysosomal iron and/or mitochondrial cytochrome c. In addition, after 72 h OTA exposure, HK-2 cells up-regulated the transcription of CAT, GSR, and SOD, which are important antioxidant enzymes required for the detoxification of ROS and regeneration of GSH. G6PD and GPX mRNA levels in LLC-PK1 cells were the only genes affected after 72 h OTA treatment by down-regulating its transcription, which may indicate that longer OTA exposure time is required to observe an adverse effect in LLC-PK1 cells. Nonetheless, whether the down-regulation of G6PD and GPX mRNA levels by OTA involved a direct mechanism, or whether they are being affected indirectly (e.g., tumor suppressor p53 and/or selenium deficiency) remains to be elucidated. In conclusion, the results presented in this study indicate that OTA, within its water solubility range, may be involved in the development of oxidative stress in HK-2 cells, as indicated by an increase in ROS levels, depletion of GSH, and increased sensitivity to cellular defense against oxidative stress, such as up-regulation of CAT, GSR, and SOD1 mRNA levels. Based on the results, it is concluded that an immortalized proximal tubule epithelial cell line from normal adult human kidney (HK-2) can offer a sensitive in vitro system for studying OTA-induced oxidative stress, while pigs are known to be the most sensitive species to OTA. To the best of our knowledge, this is the first study demonstrating a differential response of two cell lines, LLC-PK1 and HK-2, toward OTA-mediated oxidative stress.

## 5. Materials and Methods

### 5.1. Chemicals

OTA, 3-(4,5-Dimethyl-2-thiazolyl)-2,5-diphenyl-2H-tetrazolium bromide (MTT), sodium dodecyl sulfate (SDS), *N*,*N*-dimethylformamide (DMF), reduced *L*-glutathione (GSH), tert-butyl hydroperoxide (TBHP), hydrogen peroxide (H_2_O_2_), protease inhibitor cocktail, and 5,5′-dithio-bis(2-nitrobenzoic acid) (DTNB) were purchased from Sigma-Aldrich (St. Louis, MO, USA). Medium M199, keratinocyte-SFM (1×) growth medium supplied with bovine pituitary extract and human recombinant epidermal growth factor, penicillin–streptomycin (10,000 U/mL–10,000 μg/mL), Dulbecco’s phosphate buffer (no calcium and magnesium, DPBS), fetal bovine serum (FBS), 0.25% *w/v* trypsin 0.53 mM EDTA, 0.05% *w/v* trypsin 0.53 mM EDTA, 6-carboxy-2′,7′-dichlorodihydrofluorescein diacetate (carboxy-H_2_DCFDA), M-PER mammalian protein extraction reagent, Pierce bicinchoninic acid protein assay kit (BCA), TRIzol reagent, Quant-iT RiboGreen RNA assay kit, and high-capacity cDNA reverse transcription kit were obtained from Invitrogen (Waltham, MA, USA). RQ1 RNase-free DNase was obtained from Promega (Madison, WI, USA).

### 5.2. Cell Culture

Pig renal proximal tubular cell line (LLC-PK1) and human renal proximal tubular cell line (HK-2) were obtained from American Type Culture Collection (ATCC, Manassas, VA, USA) and were cultivated according to ATCC culture method recommendations. LLC-PK1 cells were cultured in M199 growth medium containing 1% *v/v* penicillin–streptomycin and 3% (*v/v*) FBS; HK-2 cells were cultured in keratinocyte-SFM growth medium supplemented with epidermal growth factor, bovine pituitary extract, and 1% (*v/v*) penicillin–streptomycin. All cells were maintained at 37 °C in a humidified atmosphere with 5% CO_2_. All cells were seeded in each specific assay plate at a density of 3.13 × 10^4^ cells/cm^2^. OTA was dissolved in pure methanol (24.76 mM) and further diluted in each cell growth medium under serum-free conditions; methanol concentration did not exceed 0.1% (*v/v*) in the final assay.

### 5.3. Cell Viability Assay

Cell viability was determined using the MTT assay, which is a widely accepted method for enumerating viable cells [90]. Briefly, cells were seeded in 96-well plate and allowed to grow for 24 h and were then treated with increasing concentrations of OTA (1.95 nM to 3 μM) in serum-free medium for different time intervals (24 h, 48 h, and 72 h). After OTA exposure, MTT was added directly to the medium (final concentration of 0.5 mg/mL), and the plate was incubated for 4 h at 37 °C. The formazan crystals formed were dissolved with 20% (*w/v*) SDS in DMF (pH 4.7), and the absorbance was read at 570 nm on a Synergy 2 microplate reader (Biotek, Inc., Winooski, VT, USA). The cell viability was calculated with respect to non-treated cells as the control, and thus, the results are expressed as percentage of control. The percentage of cell viability was calculated by the following formula: % cell viability = (Abs_sample_ − Abs_blank_)/(Abs_control_ − Abs_blank_) × 100, where Abs = absorbance value.

### 5.4. Intracellular ROS Analysis

The redox state of the cells was assessed using carboxy-H_2_DCFDA conversion to fluorescent 2′,7′-dichlorofluorescein (DCF) [91]. Briefly, cells were seeded in black and clear bottom 96-well plate in growth medium and allowed to grow for 24 h; then the medium was removed and cells washed with serum- and phenol red-free medium. Next, cells were incubated with 100 μM carboxy-H_2_DCFDA in serum- and phenol red-free medium for 30 min at 37 °C. Carboxy-H_2_DCFDA was removed by washing the cells once with serum- and phenol red-free medium, and then exposed to OTA. The fluorescence of the cells from each well was measured and recorded during several time intervals in a Synergy 2 microplate reader. The excitation filter was set at 485 ± 20 nm, and the emission filter was set at 528 ± 20 nm. OTA concentrations were selected based on the IC_50_ value at 72 h. Hydrogen peroxide and TBHP were used as positive control and their concentrations were selected based on each IC_50_ value at 6 h (data not shown). The results are expressed as the percentage increase in fluorescence and were calculated by the formula: [(F_tf_ − F_t0_)/F_t0_ × 100], where F_tf_ = fluorescence at time *f* h, and F_t0_ = fluorescence at time 0 h.

### 5.5. Glutathione (GSH) Measurement

Reduced glutathione was determined spectrophotometrically by quantifying the oxidation product (5′-thio-2-nitrobenzoic acid) upon the reaction between GSH and DNTB [92]. Briefly, cells were seeded in 6-well culture plates and allowed to grow for 24 h, culture medium was removed, and then cells were exposed to OTA, H_2_O_2_, and TBHP as in Section 5.4. After each exposure time (6 h, 24 h, 48 h, or 72 h), medium was removed, and cells were lysed directly with M-PER supplemented with 1% (*v/v*) protease inhibitor cocktail according to manufacturer’s instruction. Reduced glutathione concentrations were calculated based on a GSH standard curve using liner regression and were normalized to protein content determined by Pierce BCA protein assay. The results are expressed as percentage of control from each exposure time.

### 5.6. Real-Time Quantitative Reverse Transcription PCR (RT-qPCR)

All cells were seeded in a 100 mm culture dish (Corning, Oneonta, NY, USA) and allowed to grow for 24 h before treatment with OTA, H_2_O_2_, and TBHP as in Section 5.4. Cells were washed with DPBS, and RNA was extracted using the TRIzol reagent, following the manufacturer’s instructions. Total RNA was quantified fluorometrically using the Quant-iT RiboGreen RNA assay kit according to the manufacturer’s procedure. Genomic DNA was removed from 1 μg of RNA using RQ1 RNase-free DNase according to the manufacturer’s instructions. First-strand cDNA was synthesized from 1 μg of RNA (gDNA-free) using a high-capacity cDNA reverse transcription kit according to manufacturer’s procedure, and 10 ng of cDNA for gene expression analysis was used. The PCR components mixes were then prepared by combining each cDNA sample with 20× TaqMan gene expression assay, 2× TaqMan gene expression master mix, and RNase-free water. Quantitative RT-PCR was done by the use of StepOnePlus Real-Time PCR System (Applied Biosystems, Waltham, MA, USA). Gene expression was quantified using the ^ΔΔCt^ method, and fold-change values are reported as 2^−(ΔΔCt)^ [93]. The TaqMan gene expression assays for LLC-PK1 cells used were SOD1 (Ss03375614_u1), CAT (Ss04323025_m1), GPX1 (Ss03383336_u1), GSR1 (AJRSADI), G6PD (AJS08JQ), and GAPDH (Ss03374854_g1). The TaqMan gene expression assays for HK-2 cells used were SOD1 (Hs00533490_m1), CAT (Hs00156308_m1), GPX1 (Hs00829989_gH), GSR (Hs00167317_m1), G6PD (Hs00166169_m1), and GAPDH (Hs99999905_m1). The relative amount of each target gene was normalized to GADPH.

### 5.7. Statistical Analysis

Statistical calculations were performed using the Statistical Package for Social Sciences version 23.0 (SPSS Inc., Chicago, IL, USA). The effect of different concentrations of OTA on individual parameters was analyzed by analysis of variance (ANOVA) and Dunnett’s post hoc test, and comparisons of means was done with Tukey’s post hoc test for multiple comparison, or with Student’s *t*-test for two-group comparisons. Data represent means ± standard deviation (SD) of three independent experiments of subsequent cell passages. Differences were accepted as statistically significant at *p* < 0.05.

## Figures and Tables

**Table 1:** Pig (LLC-PK1) and human (HK-2) proximal tubule epithelial cells.

Cell line	IC_50_ (μM OTA)		
	24 h	48 h	72 h
LLC-PK1	>3	2	1
HK-2	2	0.382	0.125

## Data Availability

The data presented in this study are available in article here.
